# How to Prevent Colds

**Published:** 1875-11

**Authors:** 


### HOW TO PREVENT COLDS.

If people were blessed with common sense and a little wholesome self-denial, they might often escape severe colds and fevers by resolute measures adopted in season.

There is probably not a man, woman, or child, who is not as often as once a year afflicted with a severe cold, which ends in a cough or catarrh; and thousands there are who die every year of consumption, brought on by taking cold. He, then, who should discover a certain and effectual remedy for this complaint would be justly regarded as one of the greatest benefactors of the age. The writer does not profess to have discovered such a remedy, but he wishes to attest the truth of the following certain and effectual expedient for preventing a cold. A cold can not be instantly cured; but if it can be prevented, it is of no importance to know how it may be cured.

A bad cold, like measles or mumps, or other similar ailments, will run its course of about ten days, in spite of what may be done for it, unless remedial means are employed within forty-eight hours of its inception. Many a useful life may be spared to be increasingly useful, by cutting a cold short off in the following safe and simple manner. On the first day of taking a cold, there is a very unpleasant sensation of chilliness. The moment you observe this, go to your room and stay there. Keep it at such a temperature as will entirely prevent this chilly feeling, even if it requires 100 degrees of Fahrenheit. In addition to this, put your feet in water half-leg deep, as hot as you can bear it, adding hot water from time to time, for a quarter of an hour, so that the water shall be hotter when you take your feet out than when you put them in. Then dry them thoroughly, and put on thick, warm, woollen stockings, even if it be summer—for summer colds are most dangerous—and for twenty-four hours eat not an atom of food, but drink as largely as you desire of any kind of warm tea; and, at the end of that time, the cold will be entirely broken, without any medicine whatever. Efficient as the above means are, not one in a thousand attends to them,—led on, as most men are, by the hope that a cold will pass away of itself. Nevertheless, this article will pass under the eye of some who do not choose to run the double risk of taking physic and dying too. The expedient is a severe one for epicures and gluttons, but most persons will find it easier to fast one day than to be sick a fortnight. Sometimes fasting for three or four meals is sufficient; but the whole remedy is better than a part. Let those who are often afflicted with colds—ministers, students, and consumptives generally,—remember the above directions; if faithfully followed, they will do more good than all the pulmonics, cold-cordials, and other hurtful nostrums in the world.

---

It is the duty of parents and those intrusted with the care of children, to instruct themselves in the best method of proceeding, under the sudden diseases and dangers to which children are the most liable, as convulsions, choking, wounds, profuse bleeding, accidents from fire, water, and other casualties.

